# Effect of peer-distributed HIV self-test kits on demand for biomedical HIV prevention in rural KwaZulu-Natal, South Africa: a three-armed cluster-randomised trial comparing social networks versus direct delivery

**DOI:** 10.1136/bmjgh-2020-004574

**Published:** 2021-07-26

**Authors:** Maryam Shahmanesh, T Nondumiso Mthiyane, Carina Herbsst, Melissa Neuman, Oluwafemi Adeagbo, Paul Mee, Natsayi Chimbindi, Theresa Smit, Nonhlanhla Okesola, Guy Harling, Nuala McGrath, Lorraine Sherr, Janet Seeley, Hasina Subedar, Cheryl Johnson, Karin Hatzold, Fern Terris-Prestholt, Frances M Cowan, Elizabeth Lucy Corbett

**Affiliations:** 1 Institute for Global Health, University College London, London, UK; 2 Africa Health Research Institute, Durban, Kwa-Zulu Natal, South Africa; 3 MRC International Statistics and Epidemiology Group, London School of Hygiene & Tropical Medicine, London, London, UK; 4 London School of Hygiene and Tropical Medicine, Faculty of Epidemiology and Population Health, London, London, UK; 5 Faculty of medicine, University of Southampton, Southampton, Hampshire, UK; 6 Department of Global Health &Development, London School of Hygiene and Tropical Medicine, London, UK; 7 South African National Department of Health, Pretoria, South Africa; 8 HIV, Hepatitis and STI Department, World Health Organisation, Geneva, Switzerland; 9 Population Services International, Washington, District of Columbia, USA; 10 Department of Global Health and Development, London School of Hygiene and Tropical Medicine, London, London, UK; 11 Centre for Sexual Health HIV/AIDS Research (CeSHHAR) Zimbabwe, Harare, Zimbabwe; 12 Department of International Public Health, Liverpool School of Tropical Medicine, Liverpool, Liverpool, UK; 13 Department of Clinical Research, London School of Hygiene and Tropical Medicine, London, UK; 14 TB-HIV Group, Malawi-Liverpool-Wellcome Trust Clinical Research Programme, Blantyre, Malawi

**Keywords:** HIV, cluster randomized trial, other diagnostic or tool, public health, prevention strategies

## Abstract

**Study objective:**

We investigated two peer distribution models of HIV self-testing (HIVST) in HIV prevention demand creation compared with trained young community members (peer navigators).

**Methods:**

We used restricted randomisation to allocate 24 peer navigator pairs (clusters) in KwaZulu-Natal 1:1:1: (1) standard of care (*SOC):* peer navigators distributed clinic referrals, pre-exposure prophylaxis (PrEP) and antiretroviral therapy (ART) information to 18–30 year olds. (2) *peer navigator direct distribution (PND):* Peer navigators distributed HIVST packs (SOC plus two OraQuick HIVST kits) (3) *incentivised peer networks (IPN):* peer navigators recruited young community members (seeds) to distribute up to five HIVST packs to 18–30 year olds within their social networks. Seeds received 20 Rand (US$1.5) for each recipient who distributed further packs. The primary outcome was PrEP/ART linkage, defined as screening for PrEP/ART eligibility within 90 days of pack distribution per peer navigator month (pnm) of outreach, in women aged 18–24 (a priority for HIV prevention). Investigators and statisticians were blinded to allocation. Analysis was intention to treat. Total and unit costs were collected prospectively.

**Results:**

Between March and December 2019, 4163 packs (1098 SOC, 1480 PND, 1585 IPN) were distributed across 24 clusters. During 144 pnm, 272 18–30 year olds linked to PrEP/ART (1.9/pnm). Linkage rates for 18–24-year-old women were lower for IPN (n=26, 0.54/pnm) than PND (n=45, 0.80/pnm; SOC n=49, 0.85/pnm). Rate ratios were 0.68 (95% CI 0.28 to 1.66) for IPN versus PND, 0.64 (95% CI 0.26 to 1.62) for IPN versus SOC and 0.95 (95% CI 0.38 to 2.36) for PND versus SOC. In 18–30 year olds, PND had significantly more linkages than IPN (2.11 vs 0.88/pnm, RR 0.42, 95% CI 0.18 to 0.98). Cost per pack distributed was cheapest for IPN (US$36) c.f. SOC (US$64). Cost per person linked to PrEP/ART was cheaper in both peer navigator arms compared with IPN.

**Discussion:**

HIVST did not increase demand for PrEP/ART. Incentivised social network distribution reached large numbers with HIVST but resulted in fewer linkages compared with PrEP/ART promotion by peer navigators.

**Trial registration number:**

NCT03751826.

**Key questions**

**What is known?**

- Randomised controlled trials in Southern Africa have shown that HIV self-testing (HIVST) increases the knowledge of HIV status in adolescents and men and can improve linkage to HIV care and voluntary male medical circumcision when delivered with enablers such as financial incentives.

**What are the new findings?**

- HIVST rapidly reached men and women aged 18–30 in rural KwaZulu-Natal. However, HIVST did not create demand for biomedical HIV prevention, including Pre-Exposure Prophylaxis, compared with peer-led community-based support.

**What do the new findings imply?**

- These findings suggest that incentivised peer network models may reach young people with HIV testing. However, it would need to be provided alongside trained peer-led activities to effectively attract and engage young people into novel biomedical HIV prevention.

## Introduction

In 2019, South Africa (SA) had 7.5 million people living with HIV (PLHIV) with an estimated 200 000 new infections, mainly in adolescents girls and young women^1^ despite highly efficacious biomedical HIV prevention options, including pre-exposure prophylaxis (PrEP), voluntary medical male circumcision (VMMC) and HIV treatment with antiretroviral therapy (ART). ART improves health and eliminates onward transmission from PLHIV once sustained viral load suppression is achieved.^2 3^ PrEP can reduce HIV acquisition by up to 90%, and VMMC reduces HIV acquisition in men by 60%.^4 5^

Adolescents and youth, although highly vulnerable to HIV, have numerous structural and social barriers to taking up both HIV testing and subsequent HIV prevention services.^6–11^ HIV self-testing (HIVST), delivered as simple oral fluid or blood-based self-test kits, provides a discreet and convenient way for individuals to collect their own specimens and interpret their own results in private, overcoming testing barriers.^12–14^ HIVST can increase uptake of HIV testing among high-risk populations including young people,^15–18^ but the extent to which HIVST can create demand for subsequent HIV prevention services remains unclear.^19 20^

Realising the potential of biomedical prevention methods, including PrEP, to alter the course of the HIV epidemic in SA will require high coverage among those at risk, including adolescent girls and young women.^21 22^ This was particularly the case in KwaZulu-Natal (KZN), where, prior to this study, annual HIV incidence was 8% among women aged 20–24% and 5% among women aged 15–19.^23 24^ Meanwhile, we and others had shown that peer-led community-based approaches foster social networks and norms that endorse HIV prevention, particularly among adolescents and young people.^10 25–29^

We hypothesised that distribution of oral HIVST kits could enhance peer-led community-based health promotion strategies to mobilise demand for biomedical HIV prevention by empowering young people to test themselves in private and evaluate their candidacy for HIV care and prevention.^7 12 17 30 31^ We also hypothesised that mobilising social networks to distribute HIVST using incentivised peer-led methods (respondent-driven sampling) would extend the reach of HIVST and demand for PrEP among young people who were most at-risk compared with direct distribution.^19 32 33^

The aim of this cluster-randomised controlled trial (cRCT) was to investigate whether HIVST delivered by peers, either directly or through incentivised social networks, would increase demand for PrEP/ART among adolescent girls and young women aged 18–24 years and all young people (aged 18–30) in a rural setting with a high burden of HIV in KZN, SA.

## Methods

### Study design, setting and participants

The trial design has been described in detail elsewhere.^34^ Briefly, between March and December 2019, we conducted a three-arm cRCT comparing two models of peer delivery of HIVST (incentivised peer networks (IPN) and direct distribution by trained area-based peer navigators) with standard of care (SOC, referral by peer navigators to HIV testing, prevention and care services) for increasing the uptake of biomedical HIV prevention (PrEP) or ART among young women (18–24) and all young people (aged 18–30).

The trial was conducted in the Africa Health Research Institute (AHRI) demographic surveillance area in uMkhanyakude a poor and rural district of KZN.^35^ At the time of the trial, HIV incidence was high among young men and women (peaking at 8% per annum among women aged 20–24% and 4% per annum in men aged 25–29) and above the WHO threshold of eligibility for PrEP.^23 24^ Clinical care, including PrEP initiation, was provided through nurse-led adolescent and youth friendly study clinics. These included two accessible primary health clinics and mobile clinics that visit fixed sites across the study area. AHRI data collection clerks and nurses embedded within the 11 public health clinics serving this community also provided care to study participants.

The unit of randomisation (clusters) was 24 pairs of peer navigators working in 24 discrete areas (based on administrative divisions) of the surveillance area. Peer navigators aged 18–30 years (men and women) who had completed secondary schooling were recruited through local municipal and traditional leaders. The peer navigators underwent a 20-week training programme, which covered youth development, HIV and sexual health information, accredited HIV counselling and testing course, confidentiality, ethics and research methods, study procedures and HIVST. Competence was assessed using written and oral assessments to select 57 eligible peer navigators (24 pairs of area-based peer navigators and nine floating peer navigators (ie, on stand-by to support the arm they were randomised to)).^30^

Recruitment was community based. An estimated 12 000 young people (aged 18–30 years) residing in the 24 cluster areas were eligible to participate if able and willing to provide written informed consent, and not already taking ART. Young people were recruited by peer navigators who approached young people, in community settings, near schools and households, within each cluster to provide information and promote the study. Participants completed a brief electronic questionnaire and received a barcoded and colour co-ordinated pack that included arm-specific material and referral slips for clinical services.

Outcome ascertainment was facility based. Trained clinical research assistants screened all attendees of the three study clinics (mobile and fixed) and 11 public facilities serving the study catchment population for outcome eligibility (aged 18–30 and referred from 1 of the 24 study clusters). A screening questionnaire was administered to all consenting youth aged 18–30 years, with a request to scan their unique barcoded identifier for the clinic referral slip.

Everyone attending clinical services was offered HIV counselling and confirmatory HIV testing, immediate initiation of ART if positive or offer of PrEP (daily generic tenofovir disoproxil fumerate and emtricitabine) if HIV negative and eligible, according to South African National guidelines. Everyone received sexual health promotion, contraception and condoms. HIV-negative men were counselled on the benefits of VMMC and referred.

### Intervention

In all three arms, peer navigators promoted sexual health and the benefits of HIV testing PrEP and ART. In both intervention arms, they also demonstrated how to use the HIVST kit. All participants were asked to complete a brief check of their understanding of the information provided to them. Peer navigators recorded date of recruitment, participant’s age, area of residence and optional personal identifiers (name, national identification number and mobile phone number) and scanned the bar-coded packs for individual use (and in the case of the IPN arm for distribution). The survey took approximately 5 min to complete and was available in both English and isiZulu. Each peer navigator worked part time and recruitment continued for 6 months.

#### Standard of care

n=8 pairs of peer navigators approached young people aged 18–30 years and distributed uniquely barcoded yellow packs that included condoms and linkage information (clinic referral slips and information leaflets about HIV and PrEP).

#### Peer navigator direct distribution of HIVST

n=8 pairs of peer navigators approached young people aged 18–30 years and distributed uniquely barcoded blue HIVST packs that included SOC information and two HIVST kits (OraQuick HIV self-test kit, OraSure Technologies) with information sheets in English and IsiZulu.

#### Incentivised network distribution of HIVST

n=8 pairs of peer navigators used a modified respondent-driven sampling approach to distribute uniquely barcoded pink HIVST packs, which included SOC information and two HIVST kits. Each peer navigator recruited five 18–24-year-old female ‘seeds’ from their area. Seeds were then given up to five uniquely numbered incentivised recruitment coupons and pink HIVST packs to pass onto members of their social network. They were asked to distribute coupons and packs, demonstrate HIVST kit use and promote PrEP/ART to women aged 18–24 years preferentially but not exclusively and to avoid distribution of HIVST to those under the age of 18 or over the age of 30 years. When coupons were returned, the original individual (seed) who handed out the coupon received a sum of SAR20 (US$1.5) in mobile phone data. Each individual who returned with one of the coupons to a peer navigator (respondent) underwent the same procedure as the seeds, that is, they were given up to five uniquely numbered incentivised recruitment coupons and pink HIVST packs to pass onto members of their social network. This process continued for 6 months. For more details, see online supplemental figure 1.

10.1136/bmjgh-2020-004574.supp1 Supplementary data

### Randomisation, allocation and blinding

Prior to randomisation, pairs of peer navigators were assigned to the 24 areas they resided in. The remaining nine peer navigators were designated floating peers. Randomisation was conducted by the statistician (Thandiwe Nondumiso Mthiyani (TNM)) on the 17 January 2019. Randomisation was restricted to ensure that (1) all arms had at least one and no more than three urban areas, at least one and no more than four peri urban areas and at least two and no more than six urban areas; (2) local prevalence of HIV testing and uptake of Determined, Resiliant, Empowered, AIDS free. Mentored and Safe (DREAMS) combination HIV prevention were both within two SD of the average across the study area.^36 37^ The study statistician (TMN) generated a list of 100 000 randomisations; after the restrictions described above, 47 924 remained. The final groupings of peer navigators into three arms were completed using statistical software, into three groups of 8 pairs and three floating peer navigators (A, B and C). Allocation of peer navigator groupings to the interventions was completed at a public ceremony on 23 January 2019, where a designated group representative picked their study arm from a concealed box in the presence of the other peer navigators and the social science team.

Although the nature of the study meant that participants and peer navigators could not be blinded, masking of the investigators, statistician and the clinical (nursing) team to the allocation arm was maintained until all data were captured and cleaned at two time points: once (5 June 2019) to allow an interim analysis to inform 2019 WHO HIVST Guidelines and again at study completion (26 May 2020).

### Outcomes and measurement

The primary outcome was the number of PrEP/ART linkage events among women aged 18–24 years per peer navigator month (pnm) of outreach work. Linkage was defined as attending clinic-based PrEP eligibility screening or starting ART (based on HIV status) within 90 days of receiving a pack.

Linkage events were captured at study clinics and any of the 11 primary health clinics through scanning the barcode with the unique identifier on the clinic referral slip. Participants who reported having been given a referral slip that they had not brought with them were linked back to a peer navigator team using an algorithm based on reported colour of referral slip, pack content, residential address and identity of the peer navigator who recruited them. Furthermore, we matched their name, ID and telephone number (if available) in the eligibility questionnaire with data collected by peer navigators in the field.

Secondary study outcomes were (a) the total number of linkages among all participants (men and women aged 18–30) per pnm and (b) the total number of linkages (men and women aged 18–30) per 100 packs distributed as well as (c) the costs per HIVST distributed and young person aged 18–30 linked to care. A qualitative process evaluation is reported in a companion paper.^38^

### Analysis and sample size

We calculated the sample size for the primary outcome of linkage among women aged 18–24. Based on routine data, we estimated that one woman would link per 6 months of peer navigators’ outreach in SOC, with high potential to considerably increase linkage and that a substantial impact would be needed to justify the additional complexity and expense of adding HIVST to the peer navigator programme. There were no other data on which to base our sample size estimates, as this was the first study to address this question. We, therefore, assumed that adding HIVST would increase linkage by 100% to 150%, in which case 8 clusters per arm with 6 months of follow-up would provide 80% power to detect a 100% increase in linkage events with intercluster coefficient of variation (k) 0.25 or a 150% increase with k of 0.35.

The analysis of primary outcome followed both intention-to-treat (ITT) and per-protocol approaches. The numerator was the number of young women aged 18–24 who linked (as defined above) per cluster. In the *ITT analysis,* the denominator was the full follow-up time (months) calculated from the date at which the study started (15 March 2019) and last date of referrals (15 September 2019) per cluster. In the *per-protocol analysis,* the denominator was the actual time spent by peer navigators distributing packs in each cluster. The time worked by each peer-navigator pair in a cluster was combined to get the total time per cluster.

The analysis of secondary outcomes used ITT. For the difference in linkage/pnm, the numerator was the number of young people aged 18–30 who linked (as defined above) per cluster. Differences in linkage per 100 referral slips distributed, the numerator was the number of young people aged 18–30 who linked (as defined above) per cluster, and the denominator was the number of packs distributed by peer navigator per cluster.

Differences in rate of linkage between incentivised HIVST delivery through peer networks and direct distribution of HIVST arms were analysed using cluster-level summaries, t-tests and rate ratio. Outcomes in each cluster were summarised using rates (expressed as the number of linkage events per unit of peer navigator outreach time), with each cluster contributing a single rate to the analysis. In the analysis, the cluster-level linkage rates were log transformed due to non-normal distribution and then summarised for each study arm using means. Outcomes for both ITT and per-protocol analyses were summarised using the same approach. In each pairwise comparison, the differences between log cluster means were calculated, and t-tests were used to assess statistical significance. The cluster-level approach, although less statistically efficient than methods based on individual level regression, is considered to be more robust when there are a relatively small number of clusters.^39^ To incorporate the restriction of randomisation options used, we calculated permutation p values for the ITT analyses of the primary outcome.^40^ All analyses were performed using STATA V.15 (StataCorp LP, College Station, Texas).

### Cost analysis

Costs per pack distributed and young person linked to care were calculated using a bottom-up ingredient-based costing approach supplemented by a top-down expenditure analysis using the study budgets and expenditure reports. Capital costs included equipment (laptops for nurse and administrator, study phones and tablets for peer navigators) and training for the peer navigators (staff costs to train and supervise the peer navigators and external training). Recurrent costs included personnel costs (the peer navigators stipend, calculated per hour of work and staff to supervise the peer navigators), supplies (packs, health promotion material, referral slips, data for tablet connectivity and protective clothing), transport (including delivering packs to peer navigators in the field), Oraquick HIVST test kits, RDS incentives and other (mobile phone air time). Where costs were shared and similar for the three arms, we allocated costs equally across the three arms. This did not include the peer navigator costs that were calculated using the actual time they spent distributing referral packs. We calculated the unit cost per HIVST and referral pack distributed and the cost per person aged 18–30 linked to PrEP/ART per arm.

### Adverse events

The data were collected with written or witnessed informed consent of participants prior to being included in the study.

Provision of both HIVST and PrEP for high-risk individuals was already international and national policy within SA at the time of the trial. The independent scientific Technical Advisory Group (TAG) of the HIVST Africa initiative reviewed safety data and oversaw the trial using data reported according to a previously described adverse event (AE) reporting system for HIVST that included direct and indirect social harms from HIVST graded by severity.^41^ AEs were captured through process evaluation, community engagement units and reporting via a free hotline number provided on referral slips. Peer navigators and clinic staff logged AEs using an incident reporting form. Severe AEs were logged with the principal investigator for review and likely relationship to HIVST and reported to the STAR TAG and Ethics Review Boards.

### Patient public engagement

The primary beneficiaries of improving access to primary and secondary HIV prevention are adolescents and youth. Our patient public engagement continued throughout the research process. The study was presented to the Community Advisory Board, peer navigators and the District Department of Health, to provide input into the relevance and importance of the research question and outcome measures, before submission to Institutional Review Boards. Community-based participatory research (CBPR) was used to provide youth input into the final peer navigator interventions.^30^ Peer navigators and youth engagement during CBPR informed the design of the intervention and inclusion of the two different intervention arms, peer navigator distribution and social network distribution of the HIVST. Peer navigators witnessed randomisation and assisted in making study clinics youth-friendly, identifying the sites for the mobile clinics, and designed the information and educational materials. Peer navigators and young people who were engaged in the network distribution of HIVST recruited participants in all three arms of the study. The process evaluation explored the burden of the intervention, priorities, experiences and preferences of the adolescents and young people throughout the trial. Results dissemination included peer navigators, youth stakeholders, community advisory committee and the research community through local and international symposia.

## Results

### Participant flow and recruitment

**Figure 1:**
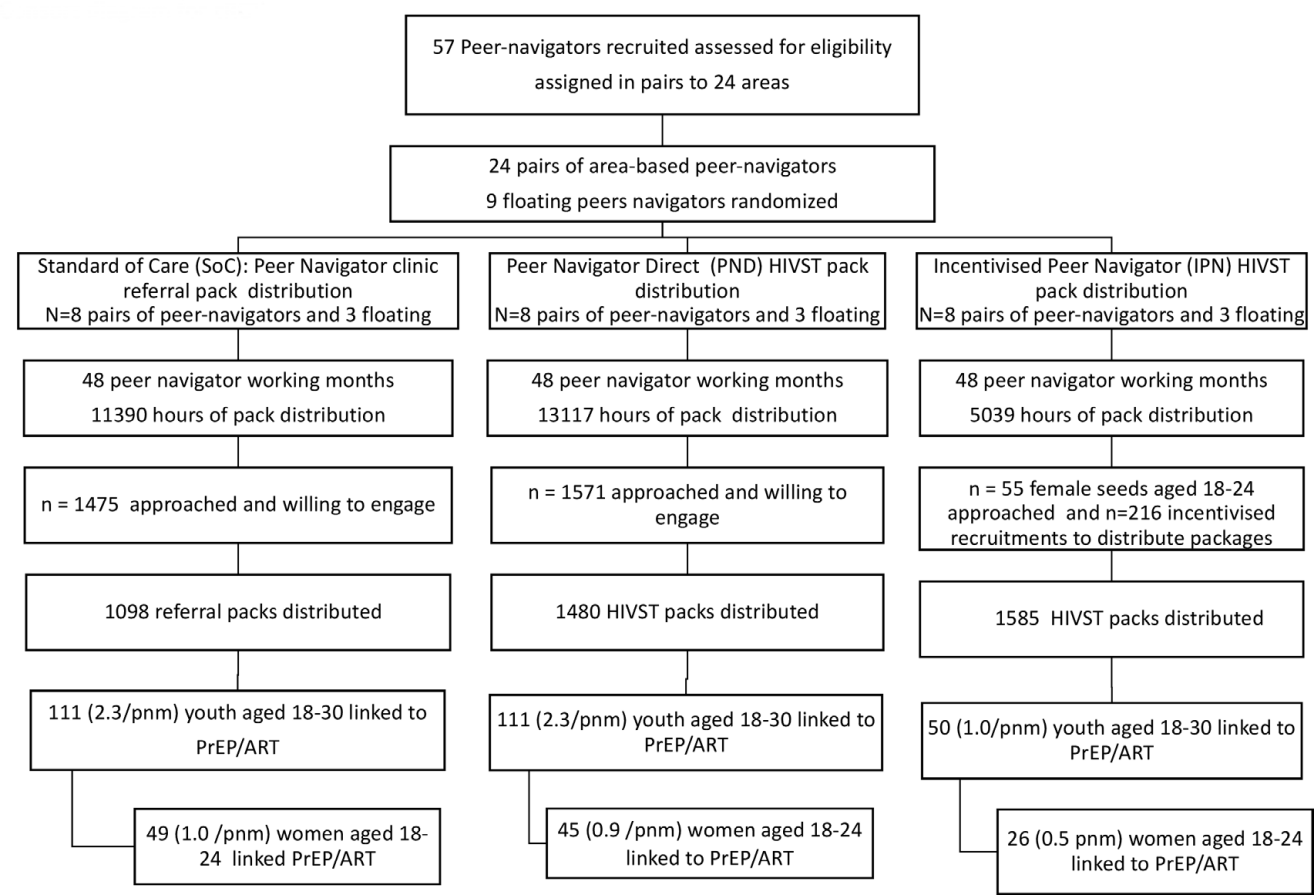
Consort diagram for cluster RCT of different models of peer-led HIVST distribution on uptake of biomedical HIV prevention and care. ART, antiretroviral therapy; HIVST, HIV self-testing; PrEP, pre-exposure prophylaxis; RCT, randomised controlled trial.

Twenty-four pairs of area-based peer navigators and nine floating peer navigators were randomly assigned to three arms (figure 1). Between 13 March 2019 and 14 September 2019, there were 144 peer navigator working months (6 months for each peer navigator pair). All 24 peer navigator pairs were retained in their originally assigned groups and included final analysis of primary and secondary outcomes (figure 1).

### Baseline data and numbers analysed

**Figure 2:**
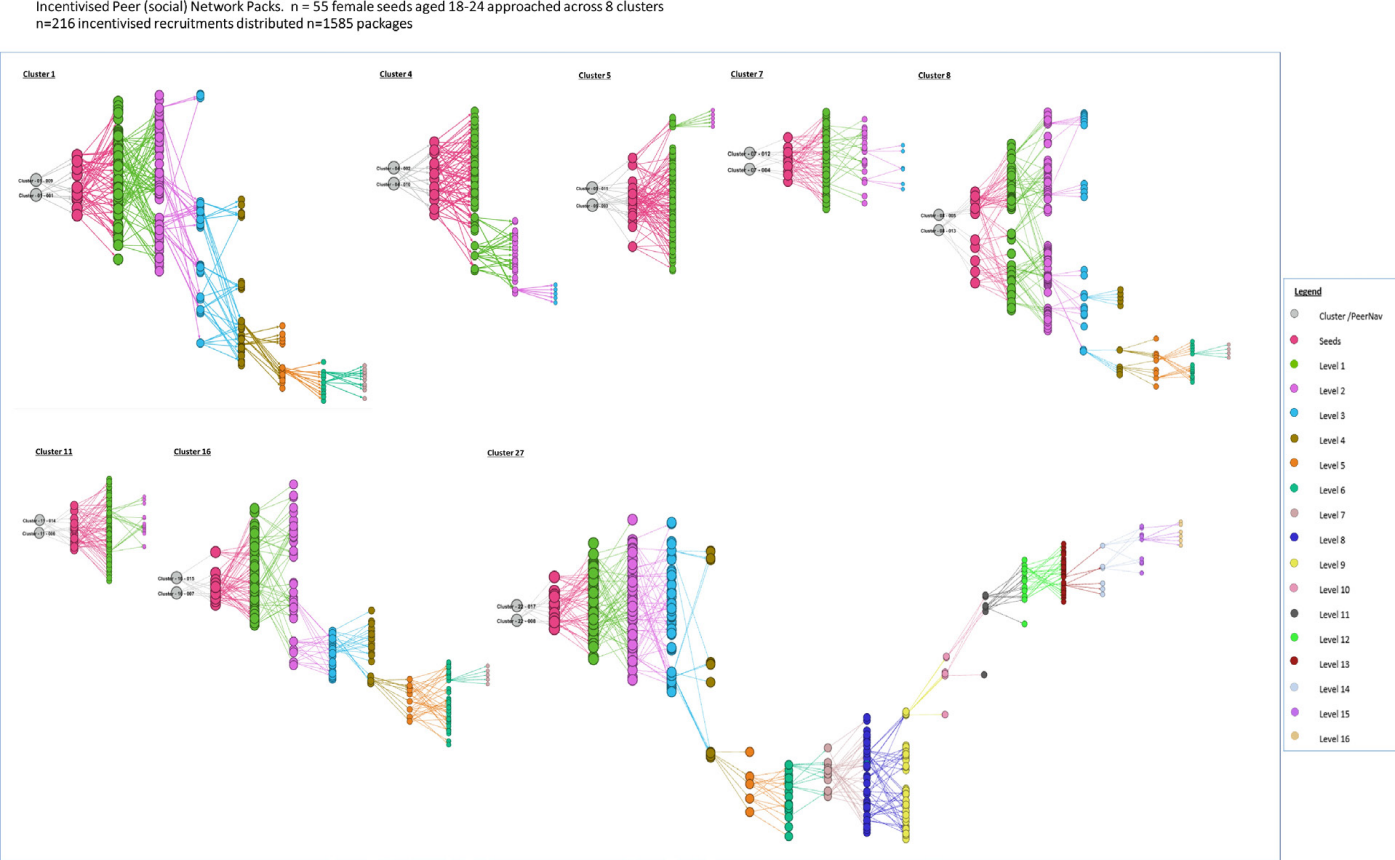
Incentivised peer navigator distribution chains (n=1585). Arm 1: incentivised peer (social) network packs. n = 55 female seeds aged 18–24 approached across 8 clusters. n=216 incentivised recruitments distributed n=1585 packages.

Between March 2019 and September 2019, 4163 referral packs were distributed across the three arms. As detailed in methods, packs included information sheets, a barcoded referral slip and, in the HIVST arms, two HIVST kits. For peer navigator direct distribution (PND) and SOC arms, numbers of referral packs directly distributed were 1480 (35.6%) and 1098 (26.4%), respectively, taking up a total of 13 117 and 11 390 hours of peer navigator time (table 1). Peer navigators in the IPN distribution arm spent less time (5039 hours total) implementing the intervention and distributed the highest number of referral packs (1585, 38.1% of total). Peer navigators in the eight IPN clusters approached n=55 seeds (women aged 18–24), this resulted in n=216 incentivised respondents during 6 months and seven recruitment waves. The structure of the social networks through which distribution occurred in the IPN arm is summarised in figure 2.

**Table 1:** Cluster level distribution of data per arm

	Incentivised peer navigator (IPN)	Peer navigator distribution +HIVST (PND)	Peer navigator no HIVST (SOC)
Number of peer navigator pairs per arm	8	8	8
Number of referral packs distributed per arm	1585	1480	1098
Mean age of pack recipients per arm	22.0	23.0	22.5
Peer navigator pair months (pnm) of work per cluster	6	6	6
Mean number of hours peer navigator pairs spent on distributing packs per cluster	629.88	1639.63	1423.75
Mean number of referral packs distributed per cluster	198.13	185.00	137.25
Mean number of 18–30 year olds linked/pnm per cluster	1.04	2.31	2.31

HIVST, HIV self-testing; IPN, incentivised peer network; PND, peer navigator direct distribution; SOC, standard of care.

As a result of the 144 pnm of work, 120 women aged 18–24 (0.83/pnm) and 272 (1.89/pnm) men and women aged 18–30 were linked to either ART or PrEP assessment (figure 1). Of these, 202 (74.3%) of the 18–30 year olds were initiated on PrEP or diagnosed with HIV and started on ART. This included 180 who were HIV negative, eligible and started PrEP.

### Linkage to PrEP/ART

Despite the higher number of referral packs distributed, fewer women aged 18–24 years linked to PrEP/ART through incentivised peer navigator (social) networks (n=26, 0.54/pnm, p=0.3) than direct peer navigator arms (PND n=45, 0.80/pnm; SOC n=49, 0.85/pnm, p=0.9), although not significantly so (table 2A). Adding HIVST did not change direct peer navigator linkage (RR 0.95 95% CI 0.38 to 2.36).

**Table 2:** Outcome of the cRCT: PrEP/ART linkage rate

A: Primary outcome: intention to treat analysis among women aged 18–24					
	Number of clients linked	Geometric mean linkage per peer navigator month of working (/pnm)	Rate ratio (95% CI)	P value*	K†
Incentivise peer navigation	26	0.54	0.68 (0.28 to 1.66)	0.40‡	0.69
Peer navigation direct	45	0.80			1.01
Incentivise peer navigation	26	0.54	0.64 (0.26 to 1.62)	0.30§	0.69
Standard of care	49	0.85			0.96
Peer navigation direct	45	0.80	0.95 (0.38 to 2.36)	0.90¶	1.01
Standard of care	49	0.85			0.96

B: Primary outcome: per protocol analysis of linkage among women aged 18–24, accounting for difference in hours spent distributing referral packs by arm					
	Number of clients linked	Geometric mean linkage per 100 hours of peer navigator (/100pnh)	Rate ratio (95% CI)	P value*	K†
Incentivise peer navigation	26	0.40	1.93 (0.95 to 3.89)	0.07	1.31
Peer navigation direct	45	0.21			0.20
Incentivise peer navigation	26	0.40	1.66 (0.75 to 3.69)	0.19	1.31
Standard of care	49	0.24			0.66
Peer navigation direct	45	0.21	0.86 (0.43 to 1.74)	0.66	0.20
Standard of care	49	0.24			0.66

C: Linkage rate among men and women aged 18–30 per peer navigator month of work (ITT)					
	Number of clients linked	Geometric mean linkage rate (/pnm)	Rate ratio	P value*	K†
Incentivise peer navigation	50	0.88	0.42 (0.18 to 0.98)	0.04	0.74
Peer navigation direct	111	2.11			0.62
Incentivise peer navigation	50	0.88	0.42 (0.18 to 1.02)	0.06	0.74
Standard of care	111	2.07			0.59
Peer navigation direct	111	2.11	1.02 (0.52 to 2.01)	0.95	0.62
Standard of care	111	2.07			0.59

D: Linkage rate among all men and women aged 18–30 per 100 referral packs distributed					
	Number of clients linked	Geometric mean linkage rate (/100 referrals)	Rate ratio	P value*	K†
Incentivise peer navigation	50	3.23	0.44 (0.21 to 0.92)	0.03	0.76
Peer navigation direct	111	7.36			0.60
Incentivise peer navigation	50	3.23	0.34 (0.14 to 0.78)	0.01	0.76
Standard of care	111	9.62			0.95
Peer navigation direct	111	7.36	0.76 (0.35 to 1.68	0.48	0.60
Standard of care	111	9.62			0.95

*P-values calculated from a t-test with a pairwise comparison of the log-transformed cluster-level means.^39^

†k=intercluster coefficient of variation.

‡Permutation p value 0.33.

§Permutation p value 0.28.

¶Permutation p value 0.89.

ART, antiretroviral therapy; cRCT, cluster-randomised controlled trial; ITT, intention-to-treat; PrEP, pre-exposure prophylaxis.

The per-protocol analysis (table 2B) used the numbers of hours spent implementing the intervention (distributing referral packs) as the denominator, rather than a fixed number of pnm. The results of this analysis suggest that linkage rates per time that peer navigators spent distributing HIVST and promoting linkage may be higher in the IPN arm (n=0.40 per 100 peer navigator hours) compared with the peer navigator arms; 0.21/100 pnh (RR 1.93 95% CI 0.95 to 3.89, p=0.07) and 0.24/100 pnh (RR 1.66 95% CI 0.75 to 3.69, p=0.2) for PND and SOC, respectively.

Table 2C shows that for all young adults aged 18–30, there was stronger evidence of lower linkage rates (0.88/pnm) for incentivised peer distribution than peer navigator distribution (2.11/pnm, RR 0.42, 95% CI 0.18 to 0.98, p=0.04) and SOC (2.07/pnm, RR 0.42 95% CI 0.18 to 1.02, p=0.06). Similarly, there was stronger evidence (table 2D) that fewer people linked to PrEP/ART per 100 packs distributed in the incentivised peer distribution (3.23/100 packs) than peer navigator distribution (7.36/100 packs, RR 0.44 95% CI 0.21 to 0.92, p=0.03) and SOC (9.62/100 packs, RR 0.34 95% CI 0.14 to 0.78, p=0.01). Adding HIVST did not change the rate of direct peer navigator linkage (p=0.5). No serious AEs or inadvertent social harms occurred. For forest plots of outcome of cRCT: PrEP/ART linkage rate, see online supplemental figure 2.

10.1136/bmjgh-2020-004574.supp2 Supplementary data

### Cost-effectiveness results

The average cost of reaching each young person with health promotion material (SOC) was less in both HIVST arms compared with SOC, reflecting higher numbers reached. Cost per kit distributed (two kits per referral pack) was US$18 for distribution through IPN compared with US$28 per HIVST distributed by peer navigators (table 3). On the other hand, the additional cost of linking each young person to PrEP/ART compared with SOC was US$114 for peer navigator direct HIVST distribution and US$513 for IPN.

**Table 3:** Cost (US$) per pack distributed and young person linked to PrEP/ART per arm

	Standard of care	Incentivised peer distribution	Peer navigator distribution
**Capital costs**			
Equipment	US$13 094	US$13 205	US$13 093
Training	US$5505	US$5505	US$5505
Total capital costs	US$18,5981	US$18 710	US$18 598
**Recurrent costs**			
Personnel costs	US$47 277	US$27 227	US$52 729
Incentives paid	0	$273	0
Oraquick test kit	0	$6781	$6444
Other supplies	$3015	$3231	$3186
Transport	$763	$438	$1173
Other recurrent	$104	$396	$181
Total recurrent costs	51 158	$38 345	$63 713
Total cost	69 757	$57 055	$82 311
**Outputs**			
Number of HIVST distributed	0	3170	2960
Number of referral pack distributed	1098	1585	1480
Number of young people aged 18–30 linked to PrEP/ART	111	50	111
**Unit costs per**			
HIVST delivered	**NA**	**$18**	**$28**
Per referral pack distributed	**$64**	**$36**	**$56**
Per young person linked to PrEP/ART	**$628**	**$1141**	**$742**

ART, antiretroviral therapy; HIVST, HIV self-testing; PrEP, pre-exposure prophylaxis.

## Discussion

In this community-based cluster-randomised trial from SA, we report the impact of two approaches to peer-based HIVST distribution on uptake of biomedical HIV prevention in adolescent girls and young women, comparing the HIVST arms against a peer-based SOC arm promoting standard HIV testing services. We found that peer-based HIVST distribution did not increase demand for PrEP/ART, despite reaching large numbers of young people. Incentivised social network distribution reached large numbers of young people with HIVST and health promotion material but resulted in fewer linkages compared with PrEP/ART promotion by peer navigators, making this one of the first trials to suggest that a direct approach by trained peers is effective in mobilising demand for biomedical HIV prevention in young people. Although incentivised social network models of HIVST distribution resulted in fewer linkages compared with direct mobilisation, peer navigators spent substantially less time interacting with young people, resulting in the highest linkage rates when peer navigator hours of implementation were used as the denominator. These findings suggest that IPN models may have a place in distributing HIV testing, if used alongside trained peer-led activities to attract and engage young people with HIV prevention.

We found that oral-based HIVST was an efficient and acceptable way to reach young people with HIV testing in this rural setting. Peer navigators managed to distribute over 6 000 HIVST either directly or through social networks over a period of 6 months, potentially reaching the majority of the 8000 young people aged 18–30 residing in their areas with minimal social harm.^38^ Our findings from rural SA replicate findings from studies elsewhere in sub-Saharan Africa that showed that community-based distribution of HIVST is an acceptable tool to reach young people with HIV testing.^14 15 17 18^ HIVST has the potential to enable young people to explore their candidacy for HIV care and prevention in privacy.^7 12^

Disappointingly, we did not show that HIVST independently mobilised demand for biomedical prevention. While knowledge of HIV status is the gateway into the cascade of care and prevention, it may not be sufficient to link young people to care and prevention. Previous work from our and other settings has highlighted the barriers young people face in linking to HIV care and PrEP.^7 9–12 22 42^ To overcome these barriers, we chose to provide PrEP through youth-friendly sexual and reproductive health services designed to be neutral to client HIV status or gender. However, our process evaluation revealed other social barriers to PrEP uptake, such as the fear of stigma and discrimination from family members, inadvertent disclosure of sexual activity and fear of side effects.^38^ These barriers mirror those described in PrEP demonstration projects across SA and suggest that HIVST as a tool to mobilise demand for PrEP may need to be used alongside other intervention to attract and engage young people.^22^

HIVST could mobilise demand for VMMC in men.^19 43^ While all young men who linked to our clinics through the trial were referred for VMMC, our study design was not able to ascertain whether they did indeed undergo the procedure. Further work is planned to explore whether or not exposure to HIVST through the trial was associated with uptake of VMMC and to explore this model further.

To our knowledge, our trial is one of the first to suggest that peer-led interventions may mobilise demand for biomedical HIV prevention in adolescents and youth.^44–46^ There has been growing body of evidence to show the effectiveness of community-based HIV care. A meta-analysis of community healthcare workers’ role in supporting HIV treatment found that it significantly improved viral suppression (pooled OR: 1.40 95% CI 1.06 to 1.86)^47^ and more recently a multicomponent peer–mentor intervention among adolescents living with HIV in Zimbabwe showed significant improvements in virological suppression over 2 years.^48 49^

Evidence for similar peer-based interventions to support biomedical HIV prevention for young people is, however, more limited. A systematic review of reviews on the cascade of HIV prevention identified 54 peer-based interventions of which the majority was among female sex workers.^45^ Only 12 studies focused on young people; these consisted of peer education in schools or participatory learning approaches to empower young men and women to take greater control over their sexual and emotional relationships. The outcome in the majority was improvements in knowledge, sexual behaviour, condom use or HIV testing, rather than biological outcomes or engagement with biomedical interventions.^45^ Our findings on the acceptability and effectiveness of trained peer navigators to enable youth engagement with biomedical prevention suggests that area-based trained peer navigators are an untapped resource to engage young people with biomedical HIV prevention. Trials are ongoing to test the effectiveness of HIV serostatus neutral peer mentorship to reduce transmissible HIV in adolescents and youth in this setting.

### Strengths and limitations

The main strength of our study was that our evaluation of using oral HIVST to mobilise demand for biomedical HIV prevention used an area-based peer navigator intervention that could be feasibly scaled up in similar rural settings in high-HIV-incidence areas of sub-Saharan Africa. Limitations include much higher intercluster variation than expected, reducing power for our primary outcome in women aged 18–24. However, the direction of effect we found for this primary outcome was similar to the statistically significant secondary outcome among all young people aged 18–30, providing confidence that our conclusions likely apply to women aged 18–24 too. One of the challenges with trials of HIVST is ascertaining linkage to care and prevention. We used multiple methods to capture linkage events that could be linked to the peer navigator pair (cluster) from which they were recruited. This protected against inter cluster contamination; however, some linkage events could be missed. Moreover, participants may have linked to care outside of the surveillance area or may have not been identified by the data clerks when attending clinic, and so we may have underestimated the linkage to care. This would, however, have been likely to affect all arms equally and so should not affect the study findings. This was an exploratory trial where pairwise comparisons were planned from the outset. However, multiple testing can give rise to family-wise type 1 error rate that is non-nominal. This needs to be considered in interpreting these results. Finally, PrEP was a new biomedical intervention in our setting. Experience from other settings suggests that time is needed for a novel biomedical intervention to embed, especially among young women. In our trial, young men and women aged 25–30 accounted for nearly 60% of those who linked to PrEP/ART: with longer duration, we may have seen demand for PrEP increase among the younger women.

## Conclusion

Our study suggests that although HIVST reached a large number of young people, it did not mobilise demand for biomedical HIV prevention. Moreover, HIVST in conjunction with trained peer-led activities to attract and engage young people to biomedical HIV prevention performed no better than trained peer-led activities alone and was more costly. These findings suggest that HIV testing may need to be provided alongside trained peer-led activities to attract and engage young people into novel biomedical HIV prevention in southern Africa.

## Data Availability

Data are available upon request. Data can be can access and download the analytical datasets through the AHRI data repository: https://data.africacentre.ac.za. https://data.ahri.org/index.php/home
