# Supplementary material for: Effect of peer-distributed HIV self-test kits on demand for biomedical HIV prevention in rural KwaZulu-Natal, South Africa: a three-armed cluster-randomised trial comparing social networks versus direct delivery
Source: BMJ Glob Health. 2021 Jul 26;6(Suppl 4):e004574. doi: 10.1136/bmjgh-2020-004574 (PMC8317107; doi:10.1136/bmjgh-2020-004574)
Supplement: Supplementary data [file bmjgh-2020-004574supp001.pdf]

24 area-based pairs of *Thetha Nami* peer-navigators, working with ~ 12000 young people aged 18-30 were randomised into 3 arms (8 pairs per arm)

Standard of Care (SOC):

- Peer-navigators distribute referral slips and information only.
- Sexual health and HIV health promotion including PrEP
- Barcoded clinic referral slips.

Peer-navigator-distribution (PND):

- *SOC plus*
- Peer-navigators distribute HIVST packs and information directly to young people.

Incentivized-peer-networks (IPN):

- *SOC plus*
- Peer-navigators recruited 8 participants "seeds" to distribute < 5 HIVST packs and information to peers within social networks.
- The "seeds" received an incentive (20 Rand = $1.5) for each respondent who contacted peer navigators for an additional 5 HIVST packs to distribute.
