# Supplementary figures and images for: Effect of peer-distributed HIV self-test kits on demand for biomedical HIV prevention in rural KwaZulu-Natal, South Africa: a three-armed cluster-randomised trial comparing social networks versus direct delivery
Source: BMJ Glob Health. 2021 Jul 26;6(Suppl 4):e004574. doi: 10.1136/bmjgh-2020-004574 (PMC8317107; doi:10.1136/bmjgh-2020-004574)

Supplementary figure: Outcome of the cRCT:PrEP/ART Linkage rate

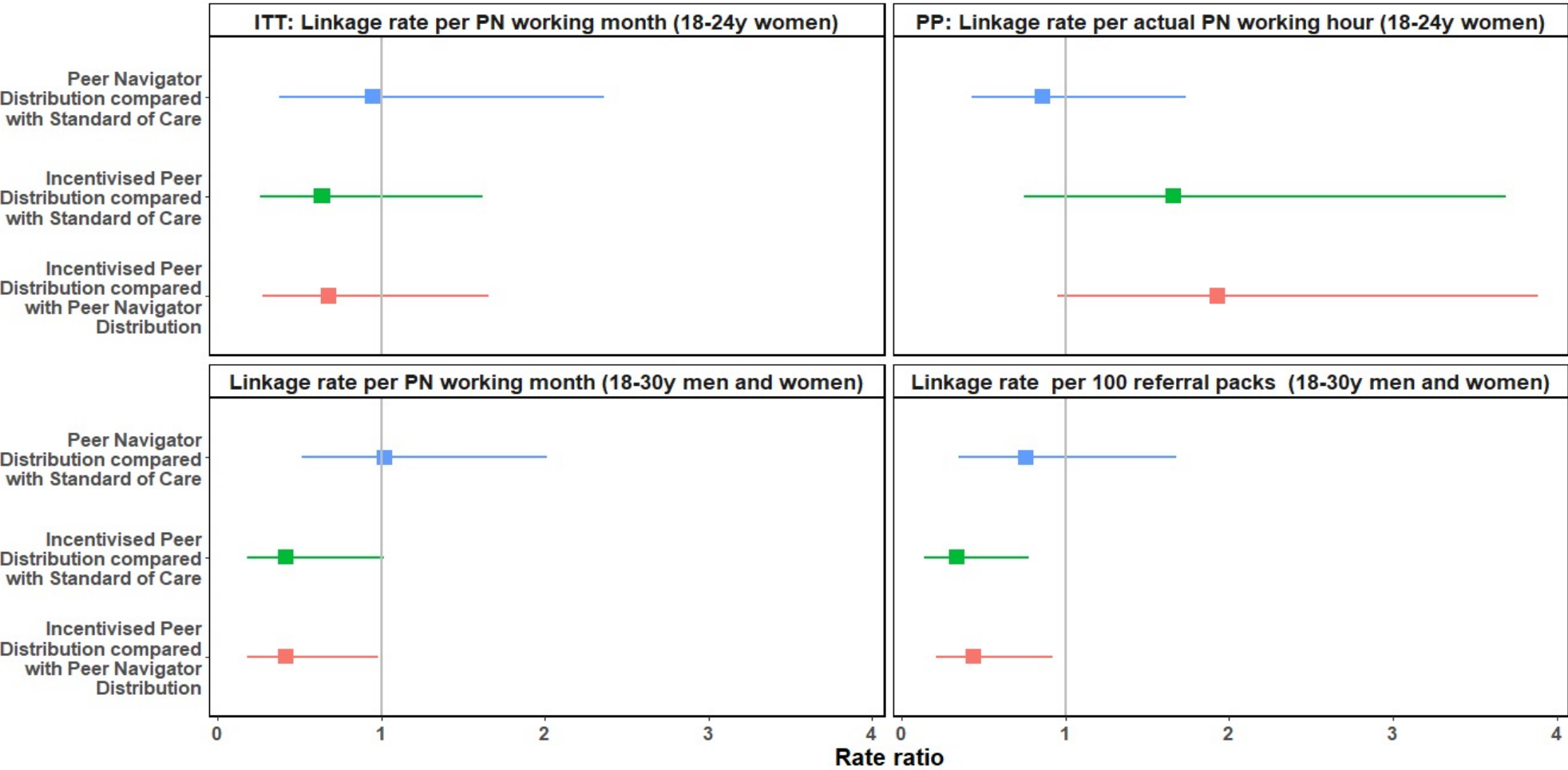

Supplement: Supplementary data [file bmjgh-2020-004574supp002.pdf]
